# A Theoretical and Practical Treatise on the Hemorrhoidal Disease

**Published:** 1885-03

**Authors:** 


**A Theoretical and Practical Treatise on the Hemorrhoidal Disease.** Giving its History, Causes, Nature, Pathology, Diagnosis and Treatment. By Wm. Bodenhamer, A. M., M. D. Illustrated by two chromo-lithographic plates and thirty-one wood-cuts. New York: Wm. Wood & Co , 56 Lafayette Place. 1884.

This work is an encyclopædic treatise on the subject. The disease denominated hemorrhoids is, perhaps, of more frequent occurrence than any other to which the human body is subject, and yet, even at the present day, much error, obscurity and confusion surround it, to the discredit and detriment of medical science, and to the encouragement of charlatanry. The distinguished author has, in this work, done much to establish the treatment of hemorrhoids upon fixed scientific principles. He gives a summary of the theory and practice of the ancients, as well as that of the modern down to the present time. His own large experience enables him to speak with authority as to the best methods of treatment, and his directions are clear, explicit and practical. It is a book which should be read by every physician.

